# Association Between Preinfarction Angina and Culprit Lesion Morphology in Patients With ST-Segment Elevation Myocardial Infarction: An Optical Coherence Tomography Study

**DOI:** 10.3389/fcvm.2021.678822

**Published:** 2022-01-18

**Authors:** Ying Wang, Zhaoxue Sheng, Jiannan Li, Yu Tan, Peng Zhou, Chen Liu, Xiaoxiao Zhao, Jinying Zhou, Runzhen Chen, Li Song, Hanjun Zhao, Hongbing Yan

**Affiliations:** ^1^Department of Cardiology, Fuwai Hospital, National Center for Cardiovascular Diseases, Peking Union Medical College, Chinese Academy of Medical Sciences, Beijing, China; ^2^China-Japan Friendship Hospital, Beijing, China; ^3^Xiamen Cardiovascular Hospital, Xiamen University, Xiamen, China; ^4^Fuwai Hospital, Chinese Academy of Medical Sciences, Shenzhen, China

**Keywords:** pre-infarction angina, ST-segment elevation myocardial infarction, optical coherence tomography, plaque rupture, lipid-rich plaque

## Abstract

**Background:**

Previous studies reported the cardiac protection effect of preinfarction angina (PIA) in patients with acute myocardial infarction (AMI). We sought to identify culprit-plaque morphology and clinical outcomes associated with PIA in patients with ST-segment elevation myocardial infarction (STEMI) using optical coherence tomography (OCT).

**Methods and Results:**

A total of 279 patients with STEMI between March 2017 and March 2019 who underwent intravascular OCT of the culprit lesion were prospectively included. Of them, 153 (54.8%) patients were presented with PIA. No differences were observed in clinical and angiographic data between the two groups, except STEMI onset with exertion was significantly less common in the PIA group (24.2 vs. 40.5%, *p* = 0.004). Patients with PIA exhibited a significantly lower incidence of plaque rupture (40.5 vs. 61.9%, *p* < 0.001) and lipid-rich plaques (48.4 vs. 69.0%, *p* = 0.001). The thin-cap fibroatheroma (TCFA) prevalence was lower in the PIA group, presenting a thicker fibrous cap thickness, although statistically significant differences were not observed (20.3 vs. 30.2%, *p* = 0.070; 129.1 ± 92.0 vs. 111.4 ± 78.1 μm, *p* = 0.088; respectively). The multivariate logistic regression analysis indicated that PIA was an independent negative predictor of plaque rupture (odds ratio: 0.44, 95% CI: 0.268–0.725, *p* = 0.001). No significant differences in clinical outcomes were observed besides unplanned revascularization.

**Conclusion:**

Compared with the non-PIA group, STEMI patients with PIA showed a significantly lower prevalence of plaque rupture and lipid-rich plaques in culprit lesion, implying different mechanisms of STEMI attack in these two groups.

## Introduction

Preinfarction angina (PIA), manifesting as an episode of angina before the onset of acute myocardial infarction (AMI), plays crucial roles in limiting reperfusion time, restricting infarct size, improving cardiac function, and reducing mortality (1–4). Recently, studies have investigated the association between clinical manifestations and culprit plaque modalities and the findings have provided insights into the mechanism of AMI. For example, a postmortems study revealed that patients with prodromal angina exhibited more plaque erosion in the culprit lesion (5) and an intravascular ultrasound study reported that the absence of PIA was related to a higher percentage of necrotic cores in the culprit site (6).

However, the association between PIA and culprit plaque characteristics in patients with ST-segment elevation myocardial infarction (STEMI) remains unclear. Optical coherence tomography (OCT) with a resolution of 10–20 μm is superior to other intravascular imaging technologies with respect to the accurate evaluation of plaque morphology and vulnerability in patients with the acute coronary syndrome (ACS) *in vivo* (7). This study identifies specific morphological characteristics of culprit plaques associated with PIA in patients with STEMI using OCT.

## Methods

### Study Population

From March 2017 to March 2019, patients aged ≥ 18 years, who were diagnosed with STEMI and underwent emergency coronary angiography and primary percutaneous coronary intervention (PCI) at Fuwai Hospital, were consecutively and prospectively enrolled in this study. After identification of the infarct-related artery using coronary angiography, OCT examinations of the culprit lesion were performed prior to interventional procedures in all the recruited patients (Fuwai Hospital OCTAMI Registry, Clinical Trials.gov: NCT03593928). STEMI was diagnosed as continuous chest pain for > 30 min, elevated biomarker levels, and an ECG manifestation of ST-segment elevation (>0.1 mV) in at least two contiguous leads or a new left bundle branch block (8). The main exclusion criteria were as follows: end-stage renal disease, contraindication to antiplatelet drugs, cardiogenic shock, significant left main coronary artery disease, or extremely tortuous or heavily calcified vessels on coronary angiography.

Preinfarction angina was defined as at least one episode of typical chest pain or referred pain that persisted for <30 min within 1 week prior to the onset of myocardial infarction (9, 10). In contrast, patients without PIA had a sudden onset of AMI without preceding angina. Unstable PIA and stable PIA were further identified based on whether the symptoms within a week were newonset/accelerated or not (4, 11). For sensitivity analysis, we defined the narrow meaning of PIA as at least two episodes of typical chest pain or referred pain that persists for 3–30 min within 1 week prior to the onset of myocardial infarction (12, 13). Clinical histories and prodromal symptom data of patients were collected and recorded in detail by the initial physician at the emergency department before primary PCI and an attending physician in the coronary care unit within 6 h after PCI. The emergency and inpatient records were retrospectively assessed within 5 days after PCI by another researcher who was blinded to the clinical, angiographic, and OCT imaging data. When there was discordance between the two records of the prodromal situation, patients were assessed again to obtain a clear classification.

### Acquisition of OCT Images

Coronary angiography was performed *via* a transradial or transfemoral approach using a 6-F or 7-F sheath. All the patients received standard antiplatelet and antithrombotic therapies according to the international guidelines (8), i.e., administration of 300 mg of aspirin (followed by 75–100 mg daily), treatment with 180 mg ticagrelor (followed by 90 mg twice daily for ≥ 12 months) or 600 mg clopidogrel (followed by 75 mg daily for ≥ 12 months), and intravascular injections of 70–100 IU/kg of unfractionated heparin prior to PCI. Glycoprotein IIb/IIIa receptor inhibitors were administered at the discretion of the operator.

The infarct-related artery was identified by at least two expert readers according to angiographic lesion morphologies, ECG manifestations, and regional wall motion abnormalities observed in the echocardiogram. Thrombus aspiration and/or gentle predilatation were used to reduce the thrombus burden and restore antegrade coronary flow. OCT images of the culprit were acquired immediately after flow restoration using the Frequency-domain Ilumien Optis OCT system and a dragonfly catheter (St Jude Medical, Westford, MA, USA). During image acquisition, continuous flushing with contrast media directly from the guiding catheter was performed to create a virtually blood-free environment.

### Analysis of OCT Images

All the OCT images were analyzed and scrutinized on a St Jude OCT Offline Review Workstation by three independent investigators who were blinded to angiographic data and clinical presentations. The first investigator was responsible for screening the suitability for culprit-plaque evaluation. The other two investigators performed qualitative and quantitative analyses of OCT images. Disagreements were resolved by consensus. The recognition of plaque morphology and parameter of OCT images were based on the validated criteria (7, 14).

**Figure 1:**
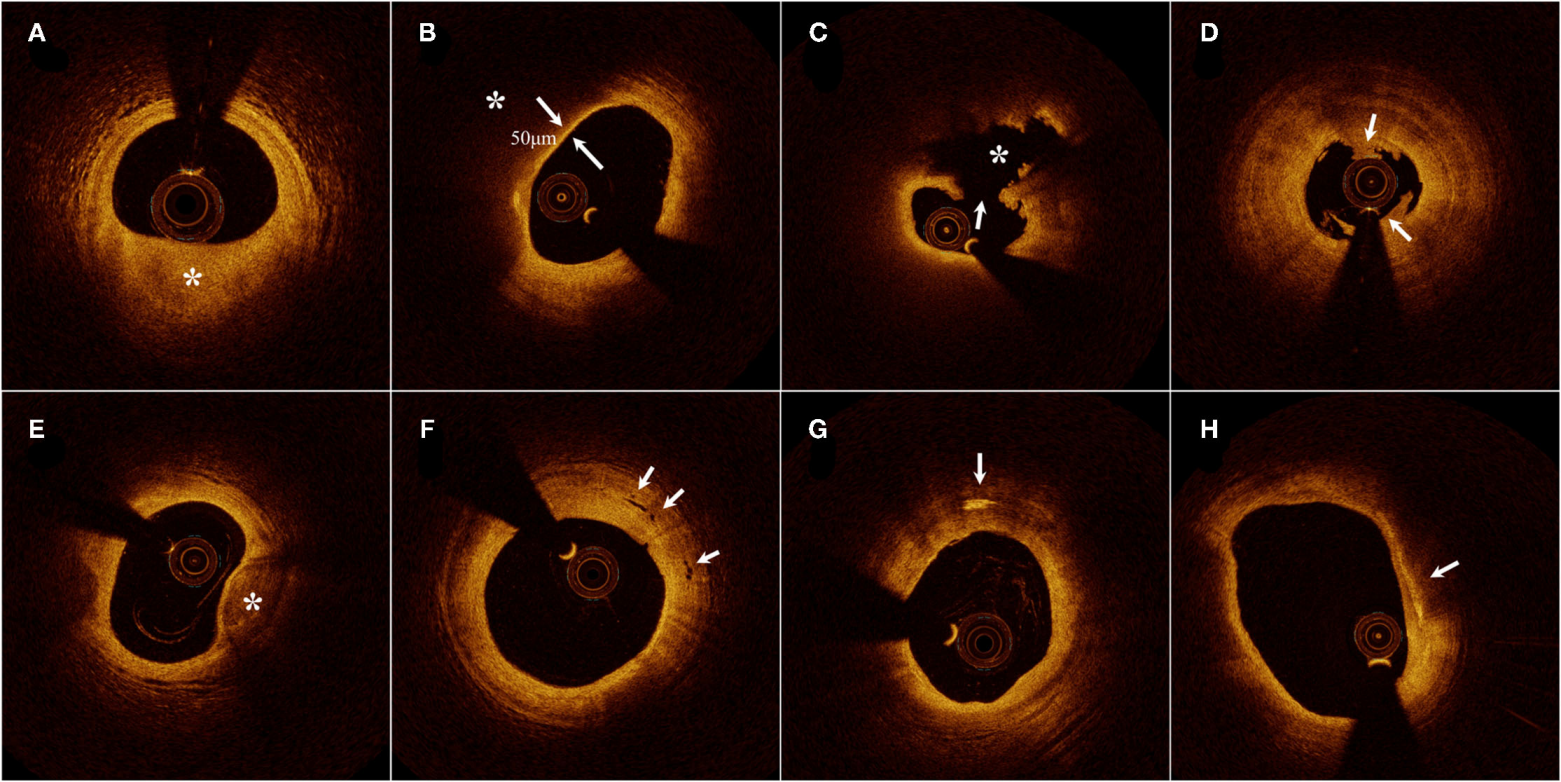
Representative cross-sectional optical coherence tomography images. **(A)** Fibrous plaque identified as a homogeneous, highly backscattering region (asterisk). **(B)** Lipid-rich plaque identified as a low-signal region with a diffuse border (asterisk) and thin-cap fibroatheroma with fibrous-cap thickness of 50 μm. **(C)** Plaque rupture identified by the discontinuous fibrous cap (arrow) and cavity formation (asterisk). **(D)** Plaque erosion identified by the presence of attached thrombus (arrow) overlying an intact plaque. **(E)** Calcification identified by the presence of a well-delineated, low-backscattering heterogeneous region (asterisk). **(F)** Microvessels recognized as low-signal, sharply delineated, and tubule luminal structures (arrow). **(G)** Cholesterol crystal (arrow) identified by linear, highly backscattering structures without remarkable backward shadowing. **(H)** Macrophage infiltration (arrow) defined as a signal-rich, highly reflective, and punctate region with backward shadowing [Adapted from reference (15) with permission].

According to the predominant component, plaques were distinguished as fibrous plaques and lipid-rich plaques (LRPs), identified as a homogeneous, highly backscattered region (Figure 1A), or a low-signal region with a diffuse border (Figure 1B). Plaque rupture was identified by the discontinuous fibrous cap with a clear cavity formation (Figure 1C), whereas plaque with an intact fibrous cap (IFC) was categorized into definite plaque erosion and probable plaque erosion according to the absence of fibrous cap disruption and the presence of thrombus. Definite plaque erosion was identified by the presence of an attached thrombus overlying an intact and recognizable plaque structure (Figure 1D); probable plaque erosion was defined as: (1) irregular luminal surface with the absence of a thrombus or (2) attenuation of underlying plaque by thrombus without superficial lipid immediately proximal or distal to the site of thrombus (16). Thin-cap fibroatheroma (TCFA) was defined as an LRP with a maximum lipid arc greater than two quadrants and a thin fibrous cap thickness (FCT) of <65 μm (Figure 1B).

Calcification within plaques was defined as the presence of well-delineated heterogeneous regions with low backscattering (Figure 1E). Microvessels were recognized as low-signal, sharply delineated, and cavity-like structures with a diameter of 50–300 μm, in more than three consecutive cross-sectional OCT images (Figure 1F). Cholesterol crystals were defined as high-signal, low-attenuating, and linear structures within the fibrous cap or plaque lipid necrosis core (Figure 1G). Macrophage infiltration was frequently found at the boundaries between the fibrous cap and inner lipid core, and was identified as signal-rich, highly reflective, punctate, or strip regions with backward shadowing (Figure 1H). An intracoronary thrombus was defined as a mass with an irregular appearance, adjacent to the luminal surface or floating within the lumen.

The quantitative OCT measurements included the following information: the length of the culprit lesion was measured from the longitudinal view; the lipid arc was measured at 1-mm intervals across the entire lesion and the largest arc was recorded; FCT was measured at the thinnest part of the fibrous cap three times, the average value was noted, and the minimal lumen area (MLA) was evaluated along the length of the target lesion.

### Clinical Outcomes and Follow-Up

The endpoint was major adverse cardiac events (MACEs) including all-cause death, recurrence of myocardial infarction (MI), stroke, and unplanned revascularization of any coronary artery. Recurrence of MI was defined by the recurrence of chest pain accompanied by either re-ST segment elevation as described above or ST-segment depression attributed to myocardial ischemia and re-elevation of troponin I > 25%. Stroke was defined as persistent neurological dysfunction with documentation of acute cerebral infarction on CT and/or MRI. Outcome data were collected by outpatient visits or telephone interviews when patients were routinely followed up at 1, 6, and 12 months after discharge. For those who survived for more than a year, a subsequent follow-up would be performed annually.

### Statistical Analysis

Continuous data, expressed as mean ± SD or median (25th and 75th percentiles), were compared using Student's *t*-test or the Mann–Whitney *U*-test. Categorical data, expressed as counts and percentages, were compared using the Chi-square test or Fisher's exact test. Interobserver and intraobserver variabilities were evaluated using kappa statistics for the qualitative variables of plaque morphologies and intraclass correlation coefficients for quantitative assessments. The logistic regression analyses with adjustments for confounding factors were used to determine the associations between the presence of PIA and plaque rupture. Survival curves were constructed using the Kaplan–Meier method and compared using the log-rank test. A two-tailed value of *p* < 0.05 was considered as statistically significant. All the statistical analyses were performed using the SPSS (version 25.0; IBM Corporation, Armonk, NY, USA) and R (http://www.r-project.org/) statistical packages.

## Results

### Baseline Characteristics

**Figure 2:**
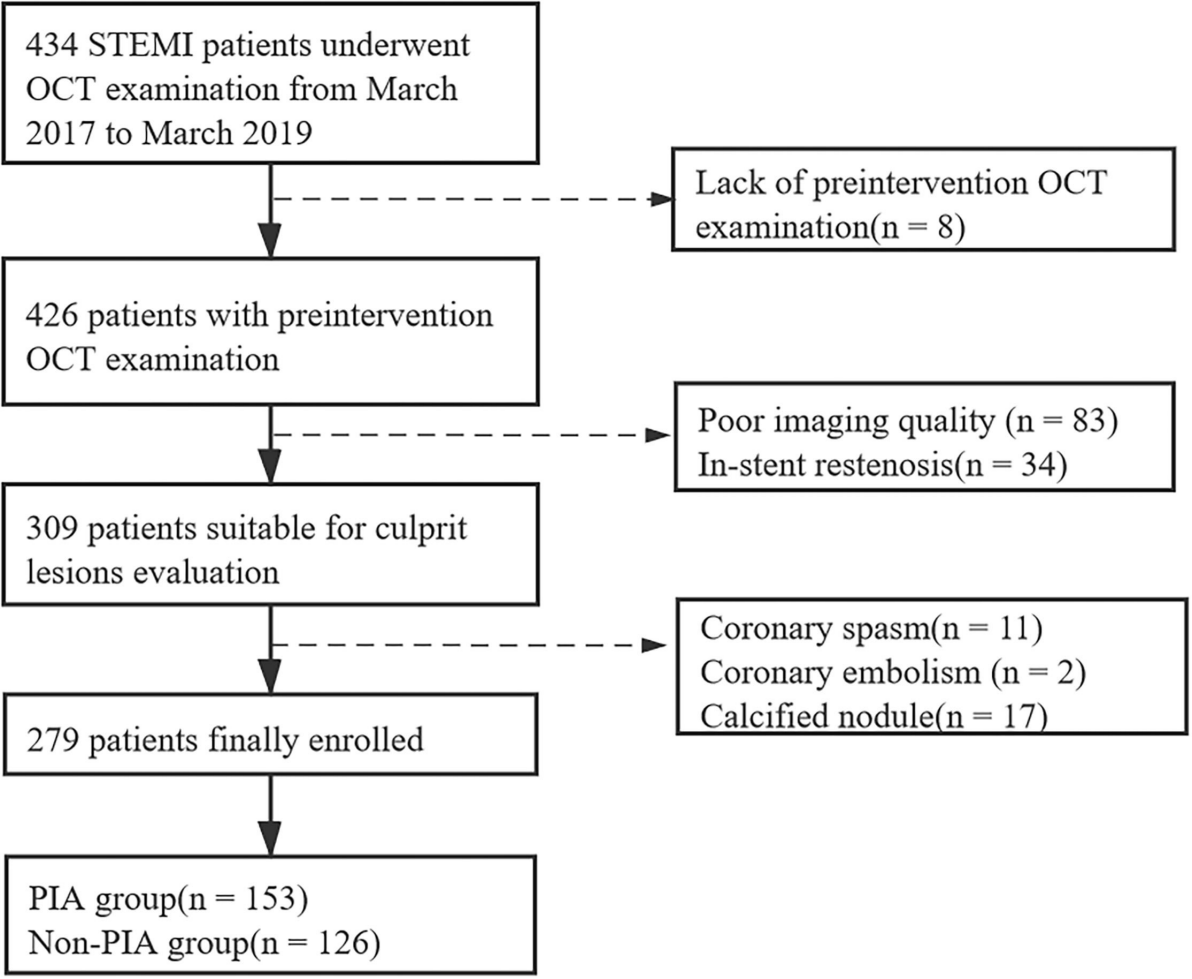
Study flowchart. STEMI, ST-segment elevation myocardial infarction; OCT, optical coherence tomography; PIA, preinfarction angina.

Among 434 patients with STEMI who underwent OCT imaging of culprit lesions, 279 eligible patients (64.3%) were enrolled in this study. Moreover, 155 patients who underwent OCT were excluded for the following reasons: lack of preintervention OCT images (*n* = 8), poor imaging quality due to massive thrombus (*n* = 83), in-stent restenosis (*n* = 34), coronary spasm (*n* = 11), coronary embolism (*n* = 2), and calcified nodule (*n* = 17). The study flowchart is shown in Figure 2. Comparisons of baseline characteristics between the included and excluded patients are given in Supplementary Table 1. The included patients had higher levels of total cholesterol (TC) and low-density lipoprotein cholesterol (LDL-C), whereas no significant differences were found in age, sex, body mass index (BMI), history of hypertension, diabetes, dyslipidemia, smoking, or other laboratory parameters.

Among the enrolled patients, 153 patients (54.8%; 125 men and 28 women) were diagnosed with PIA, whereas 126 patients (45.2%; 104 men and 24 women) had no PIA. Table 1 presents a comparison of baseline clinical characteristics between these two groups. Compared with patients without PIA, patients with PIA showed the lower peak troponin I levels [17.6 (8.6–38.4) vs. 27.0 (12.1–51.9), *p* = 0.010]. Moreover, STEMI onset with exertion was significantly less common in patients with PIA (24.2 vs. 40.5%, *p* = 0.004). No differences were observed in other clinical data.

**Table 1:** Baseline characteristics.

**Variables**	**PIA group (*n* = 153)**	**Non-PIA group (*n* = 126)**	***P*-value**
Age, years	56.8 ± 11.8	58.0 ± 11.1	0.406
BMI, Kg/m^2^	26.1 ± 4.0	26.2 ± 3.4	0.863
Men, *n* (%)	125 (81.7)	104 (82.5)	0.877
Smoking, n (%)	110 (71.9)	84 (66.7)	0.363
Attack upon exertion, *n* (%)	37 (24.2)	51 (40.5)	0.004^*^
**Medical history**, ***n*** **(%)**			
Hypertension	95 (62.1)	68 (54.0)	0.181
Dyslipidemia	137 (89.5)	116 (92.1)	0.538
Diabetes mellitus	46 (30.1)	49 (38.9)	0.129
Prior PCI	9 (5.9)	12 (9.5)	0.264
LVEF at admission, %	55.4 ± 5.8	54.6 ± 6.6	0.293
Killip grade, *n* (%)			0.591
I–II	152 (99.3)	124 (98.4)	
III–IV	1 (0.7)	2 (1.6)	
**Laboratory findings**			
White blood cells, 10^6^/L	10.4 ± 3.2	10.9 ± 3.1	0.163
Hs-CRP, mg/L	5.5 (2.7–10.4)	6.1 (2.1–11.0)	0.711
eGFR, mL/min/1.73 m^2^	99.0 (85.0–113.4)	92.9 (75.3–114.2)	0.063
HbA1c, %	6.5 ± 1.4	6.6 ± 1.6	0.419
TC, mg/dL	166.3 (141.9–194.5)	169.2 (141.1–203.8)	0.657
TG, mg/dL	122.2 (79.7–176.6)	127.1 (83.5–187.9)	0.593
LDL-C, mg/dL	104.8 (85.1–127.8)	110.2 (84.7–127.6)	0.915
HDL-C, mg/dL	41.4 (36.2–47.0)	40.4 (34.8–48.0)	0.457
Lipoprotein (a), mg/L	175.0 (75.8–389.1)	156.0 (68.0–356.5)	0.599
troponin I, ng/ml	0.8 (0.1–3.8)	1.0 (0.1–5.8)	0.360
Peak troponin I, ng/ml	17.6 (8.6–38.4)	27.0 (12.1–51.9)	0.010^*^
**Prior medications**			
Aspirin, *n* (%)	56 (36.6)	49 (38.9)	0.711
P_2_Y_12_ inhibitor, %	37 (24.2)	34 (27.0)	0.679
Statin, %	25 (16.3)	22 (17.5)	0.873

*Continuous data are presented as mean ± SD or median (25th and 75th percentile). Categorical data are presented as numbers (%)*.

* *p <0.05*.

*PIA, preinfarction angina; BMI, body mass index; PCI, percutaneous coronary intervention; LVEF, left ventricular ejection fraction; HS-CRP, high-sensitivity C-reactive protein; eGFR, estimated glomerular filtration rate; HbA1c, glycated hemoglobin; TC, total cholesterol; TG, triglyceride; LDL-C, low-density-lipoprotein cholesterol; HDL-C, high-density lipoprotein-cholesterol*.

### Procedural Data

As shown in Table 2, no significant differences were observed in the angiographic findings, such as the distribution of culprit vessels, the number of involved vessels, incidence of aspiration, predilation, door to balloon times, stent implantation, and the preintervention Thrombolysis in MI (TIMI) grade flow of ≤ 1. A total of 266 (95.3%) patients underwent stent implantation on the culprit artery, and the incidence of stent placement was higher in patients with plaque rupture than IFC but non-significant (97.1 vs. 93.5%, *p* = 0.25).

**Table 2:** Angiographic findings.

**Variables**	**PIA group (*n* = 153)**	**non-PIA group (*n* = 126)**	***P*-value**
Culprit vessels, *n* (%)			0.984
LAD	73 (47.7)	62 (49.2)	
LCX	16 (10.5)	13 (10.3)	
RCA	64 (41.8)	51 (40.5)	
LM disease	6 (3.9)	3 (2.4)	0.469
Coronary artery lesions, *n* (%)			0.557
SVD	41 (26.8)	27 (21.4)	
DVD	52 (34.0)	48 (38.1)	
TVD	60 (39.2)	51 (40.5)	
**Prior-PCI procedures**, ***n*** **(%)**			
Aspiration	97 (63.4)	76 (60.3)	0.622
Pre-dilation	122 (79.7)	100 (79.4)	0.999
Pre-TIMI flow ≤ 1	110 (71.9)	86 (68.3)	0.514
Door-to- balloon time, minutes	103.5 (76.0–138.8)	113.0 (87.0–152.0)	0.076
Stent implantation	146 (95.4)	120 (95.2)	1.000

*Data are presented as mean ± SD or number (%) or median (interquartile range)*.

*PIA, preinfarction angina; LAD, left anterior descending; LCX, left circumflex artery; RCA, right coronary artery; LM, left main coronary artery; SVD, single-vessel disease; DVD, double-vessel disease; TVD, three-vessel disease; PCI, percutaneous coronary intervention; TIMI, Thrombolysis in Myocardial Infarction*.

### Optical Coherence Tomography Findings

Comparisons of OCT characteristics between the two groups are shown in Table 3. Remarkably, patients with PIA exhibited a significantly lower incidence of plaque rupture (40.5 vs. 61.9%, *p* < 0.001) and LRPs (48.4 vs. 69.0%, *p* = 0.001) in the culprit lesions. Conversely, the prevalence of IFC was higher in patients with PIA (59.5 vs. 38.1%, *p* < 0.001). In addition, the prevalence of TCFA tended to be lower in patients with PIA than in those patients without PIA (20.3 vs. 30.2%, *p* = 0.070). Similarly, patients with PIA had a higher FCT, although statistically significant differences were not observed (129.1 ± 92.0 vs. 111.4 ± 78.1 μm, *p* = 0.088). A further *post-hoc* power analysis showed that this study is underpowered to detect a significant difference in the incidence of TCFA between these two groups (power value = 0.479, data not shown).

**Table 3:** Optical coherence tomography characteristics.

**Variables**	**PIA group (*n* = 153)**	**Non-PIA group (*n* = 126)**	***P*-value**
Plaque morphology, *n* (%)			<0.001^*^
Plaque rupture	62 (40.5)	78 (61.9)	
Intact fibrous cap	91 (59.5)	48 (38.1)	
Plaque morphology, *n* (%)			<0.002^*^
Plaque rupture	62 (40.5)	78 (61.9)	
Definite plaque erosion	71 (46.4)	37 (29.4)	
Probable plaque erosion	20 (13.1)	11 (8.7)	
Plaque type, *n* (%)			0.001^*^
Lipid-rich plaque	74 (48.4)	87 (69.0)	
Fibrous plaque	79 (51.6)	39 (31.0)	
TCFA, *n* (%)	31 (20.3)	38 (30.2)	0.070
Healed plaque	23 (18.3)	33 (21.6)	0.591
Calcification, *n* (%)	77 (50.3)	67 (53.2)	0.718
Macrophage, *n* (%)	80 (52.3)	72 (57.1)	0.469
Micro-vessels, *n* (%)	31 (20.3)	18 (14.3)	0.209
Cholesterol crystal, *n* (%)	10 (6.5)	12 (9.5)	0.380
Thrombus, *n* (%)	150 (98.0)	122 (96.8)	0.705
Minimal FCT, μm	129.1 ± 92.0	111.4 ± 78.1	0.088
Stenosis length, mm	18.0 ± 6.1	19.0 ± 6.6	0.175
Maximal lipid arc, °	302.7 ± 74.9	306.1 ± 68.3	0.693
MLA, mm^2^	1.86 ± 0.74	1.91 ± 0.68	0.633

*Data are presented as mean ± SD or number (%) or median (interquartile range). Categorical data are presented as numbers (%)*.

* *p <0.05*.

*PIA, preinfarction angina; TCFA, thin-cap fibroatheroma; FCT, fibrous cap thickness; MLA, minimal lumen area; °angle of degrees*.

**Figure 3:**
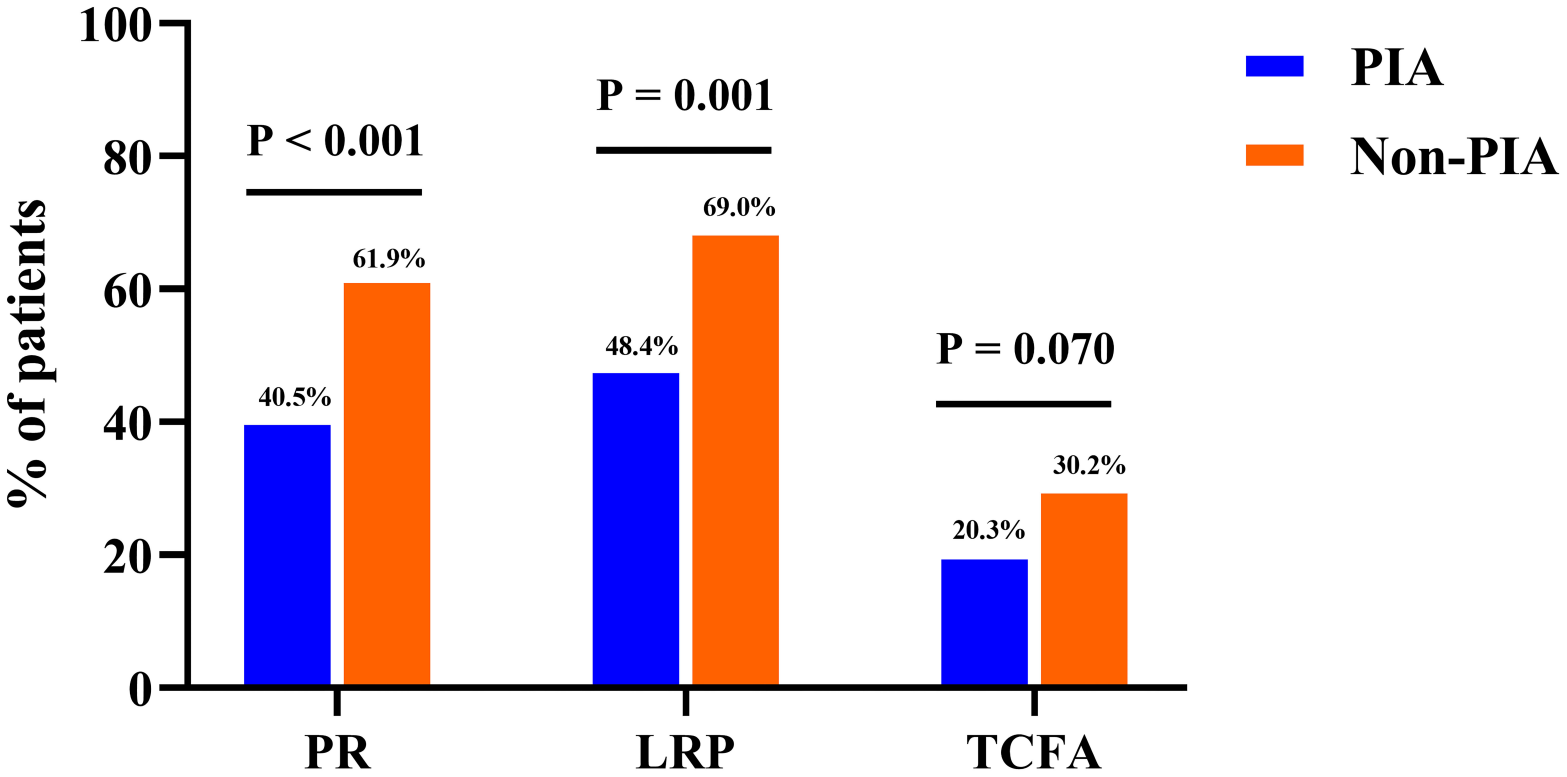
Bar graphs of optical coherence tomography findings of coronary plaques between groups. Comparisons of the incidence of plaque rupture (PR), lipid-rich plaques (LRPs), and thin-cap fibroatheroma (TCFA) showed significant differences between patients in the PIA and non-PIA groups. PIA, preinfarction angina.

Moreover, no differences were observed between patients with and without PIA in the prevalence of other microstructural features including calcification, macrophage infiltration, microvessels, cholesterol crystals, and thrombi as well as in the quantitative OCT parameters including stenosis length, maximum lipid arc, and MLA (Table 3; Figure 3). The kappa statistics of interobserver and intraobserver variability for the qualitative assessment of plaque rupture/IFC were 0.860 and 0.906, respectively. The inter-rater reliability was good for the MLA (intraclass correlation coefficient = 0.988).

Baseline characteristics, angiography, and OCT findings in patients with narrow PIA (angina lasting for 3–30 min and occurring ≥ 2 times within 1 week) or non-PIA are shown in Supplementary Table 2. Supplementary Table 3 shows the clinical, angiographic, and OCT characteristics of patients with stable PIA, unstable PIA, or non-PIA. Supplementary Table 5 shows the clinical and OCT characteristics of the PIA and non-PIA groups when patients with calcified nodules were included.

The univariate logistic regression analysis showed that the presence of PIA was closely associated with plaque rupture (Table 4). After adjusting for potential confounding factors including age, sex, BMI, hypertension, smoking, TC, triglyceride, LDL-C, high-density lipoprotein cholesterol, high-sensitivity C-reactive protein, estimated glomerular filtration rate (eGFR), and prior statin therapy, age and reduced eGFR (lower than median) were independent predictors of plaque rupture (age, odds ratio: 2.04, 95% CI: 1.23–3.36, *p* = 0.005; reduced eGFR, odds ratio: 1.76, 95% CI: 1.07–2.90, *p* = 0.027; respectively) and the presence of PIA remained negatively predictive of plaque rupture (odds ratio: 0.44, 95% CI: 0.268–0.725, *p* = 0.001).

**Table 4:** The logistic regression analysis of plaque rupture.

**Variables**	**Univariate**		**Multivariate**	
	**OR (95% CI)**	***P*-value**	**OR (95%CI)**	***P*-value**
Age	2.29 (1.42–3.70)	0.001^*^	2.04 (1.23–3.36)	0.005^*^
Men	1.49 (0.81–2.77)	0.203		
BMI	1.14 (0.71–1.82)	0.590		
PIA	0.42 (0.26–0.68)	<0.001^*^	0.44 (0.268–0.725)	0.001^*^
Diabetes mellitus	1.50 (0.91–2.47)	0.111		
Hypertension	1.01 (0.63–1.63)	0.960		
Smoking	0.85 (0.51–1.42)	0.542		
TC	0.96 (0.60–1.53)	0.857		
TG	0.90 (0.56–1.45)	0.675		
LDL-C	1.14 (0.71–1.82)	0.590		
HDL-C	1.11 (0.69–1.77)	0.676		
Hs-CRP	0.85 (0.51–1.42)	0.538		
eGFR	2.10 (1.30–3.39)	0.002^*^	1.76 (1.07–2.90)	0.027^*^
Prior statin use	1.04 (0.56–1.95)	0.894		

*Hs-CRP values were transformed into categorical data according to the value (above or below 3 mg/l). Other variables were transformed into categorical data of above-median or below-median*.

* *p <0.05*.

*OR, odds ratio; BMI, body mass index; PIA, preinfarction angina; TC, total cholesterol; TG, triglyceride; LDL-C, low-density-lipoprotein cholesterol; HDL-C, high-density lipoprotein-cholesterol; Hs-CRP, high-sensitivity C-reactive protein; eGFR, estimated glomerular filtration rate*.

### Clinical Outcomes

**Figure 4:**
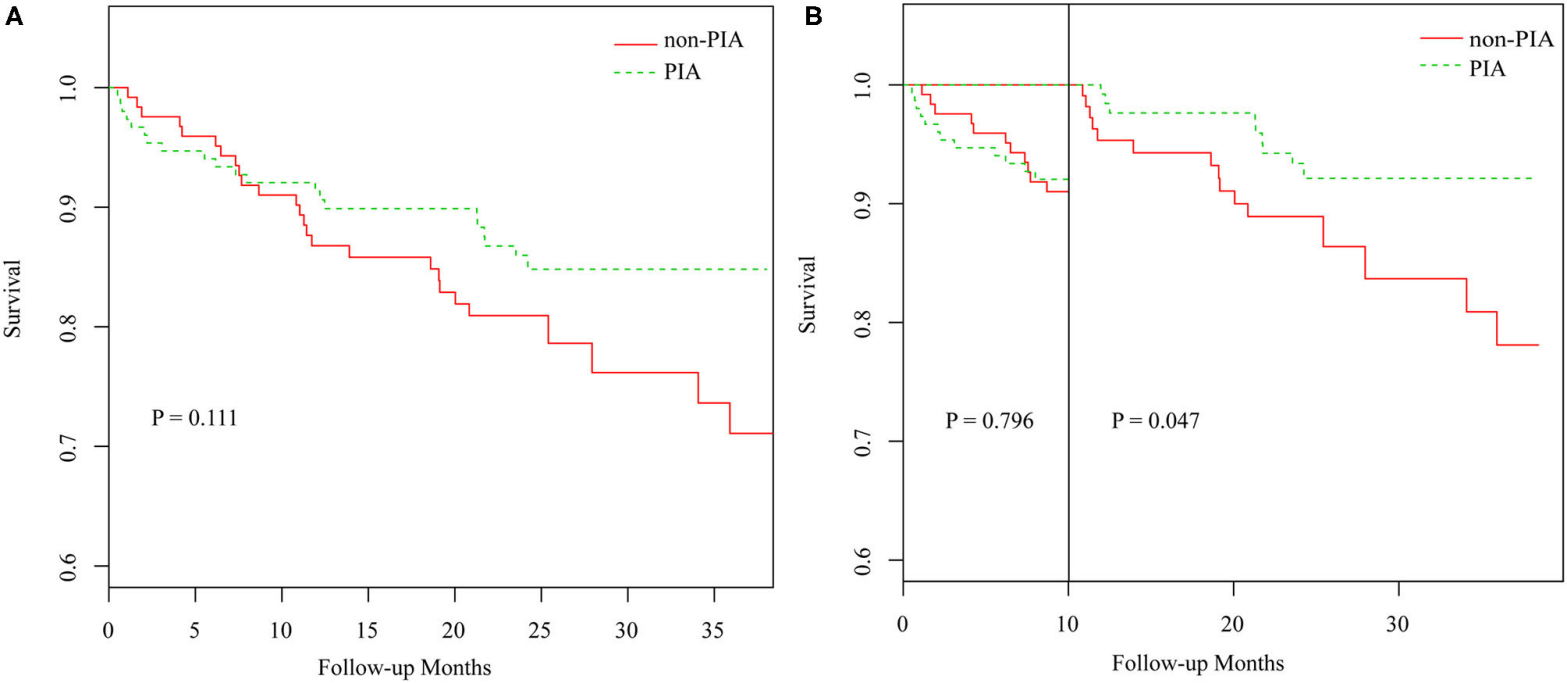
The Kaplan-Meier curves of cumulative MACE-free survival probability. **(A)** Kaplan-Meier curve of PIA and non-PIA group, **(B)** Landmark analysis for the survival rate of PIA and non-PIA group. MACE, major adverse cardiac events; PIA, preinfarction angina.

During a mean follow-up time of 22.7 months, 275 (98.6%) patients had available clinical follow-up data. No significant differences in clinical outcomes were observed, except for unplanned revascularization (Figure 4A; Supplementary Table 4). However, as shown in Figure 4B, the landmark survival analysis with the log-rank test showed that patients with PIA had a significantly higher cumulative event-free survival rate than those without PIA after 10 months (*p* = 0.047).

## Discussion

In this prospective, homogeneous, and well-defined cohort of patients with STEMI who underwent OCT imaging, we investigated the correlation between PIA and OCT findings of culprit lesion morphology. This study reveals the following key findings: (1) patients with STEMI had a high incidence of PIA; (2) patients with PIA had a significantly lower prevalence of plaque rupture or LRP in the culprit lesion than those without PIA; (3) PIA absence was an independent predictor of plaque rupture; and (4) PIA was associated with lower MACE risk at 10 months after STEMI. The present findings substantially add to the knowledge on the association between clinical manifestations and intravascular imaging.

### Prevalence of PIA

In this study, PIA occurred in 54.8% of patients with STEMI within 1 week before the onset of MI. The prevalence of prodromal chest symptoms in patients with AMI has been reported to vary between 34.8 and 50% in previous studies with different time spans for the PIA definitions (17–19), which was similar to our results. These observations demonstrate the high incidence of prodromal angina. Moreover, prodromal angina was reported to be a strong predictor of short- and long-term survival in AMI and patients with AMI without PIA tended to have a worse prognosis (20). However, despite its high incidence and prognostic value, PIA is rarely considered in current clinical trials. Thus, the clinical and prognostic value of prodromal symptoms in MI should be emphasized in further studies.

### Preinfarction Angina and Plaque Vulnerability

Patients without PIA exhibited a significantly higher incidence of plaque rupture; the finding is consistent with prior results (11, 21). Notably, the occurrence of plaque rupture is closely related to more vulnerable atherosclerosis and a worse prognosis (22). In addition, we noticed more LRPs in patients without PIA than in those with PIA. Consistently, a multicenter OCT study demonstrated that LRP, which is unstable and prone to rupture (23), could be used to predict an increased risk of cardiac events, regardless of whether it was located in the culprit region (24). Therefore, morphological findings *via* OCT in this study may reflect the greater vulnerability of plaque characteristics in patients without premonitory symptoms.

Furthermore, in terms of plaque vulnerability, TCFA identified as a low FCT (<65 μm) overlying a large LRP has been appraised as a pivotal precursor for plaque rupture (25, 26). This study found that the prevalence of TCFA tended to be lower in patients with PIA than in those without PIA, but the difference was not significant (*p* = 0.070). This discrepancy might be partly attributable to the relatively small sample size in the *post-hoc* power analysis.

In this study, the pathological morphology of plaques was rigorously assessed, although we could not exclude the potential effect of thrombus aspiration and predilatation prior to OCT imaging on the vessel wall and plaque morphology. Based on our experience, suspected rupture-like plaque caused by thrombus aspiration and predilatation could be distinguished from plaque rupture by distinct features: The latter often present with a cavity built on the necrotic core, whereas the former appear as avulsion of the intima.

### Preinfarction Angina and Physical Exertion

In this study, STEMI onset upon exertion was significantly more common in patients without PIA than in those with PIA. In line with our results, the association between the lack of premonitory symptoms and triggering by physical exertion has been proposed by previous studies (27, 28). The higher incidence of STEMI onset upon exertion in patients without previous angina may be due to the following mechanisms. First, intense physical exertion was confirmed as a trigger of AMI onset (29, 30) and was reported to be associated with a 2.31-fold increase of AMI risk in the effect of potentially modifiable risk factors associated with myocardial infarction in 52 countries (INTERHEART) study (31). A case-crossover study (32) showed that the risk of AMI onset was significantly elevated with increasing physical exertion intensity. Individuals who experience PIA for several days may be likely to have reduced voluntary physical exercise. Moreover, based on our observation, vulnerable features observed in patients without PIA and a higher incidence of MI induced by exertion may imply that patients without prodromal symptoms have a lower threshold for abrupt coronary occlusion. However, the precise mechanisms remain poorly understood. Thus, further studies are needed to clarify the underlying mechanisms.

### Preinfarction Angina and Plaque Rupture

Plaque rupture, the dominant mechanism contributing to AMI or sudden cardiac death (33), was responsible for 65% of patients admitted for STEMI, whereas 33% of patients had plaque erosion (34). Moreover, an OCT analysis in the CompariSon of Manual Aspiration with Rheolytic Thrombectomy in patients undergoing primary PCI (SMART) trial (35) showed that the incidence of plaque rupture in patients with STEMI was 58%. Consistent with those previous results, 50.3% of patients in this study showed the feature of plaque rupture, regardless of the presence or absence of premonitory angina. Importantly, patients with ruptured plaques have high-risk clinical characteristics and poor prognosis compared to those with IFC (22). Furthermore, the Effective anti-thrombotic therapy without stenting: intravascular optical coherence tomography-based management in plaque erosion (EROSION) study has revealed the safety of conservative treatment with antithrombotic therapy without stenting in patients with ACS and present with plaque erosion (36, 37), which suggested that treatment strategies are different between different pathological plaque types.

Therefore, the identification of plaque rupture is a crucial and effective method for risk stratification and management in ACS. Consistent with the findings in a small-size investigation with ACS cohort (38), this study with a larger STEMI cohort demonstrated that the absence of prodromal angina was a predictor of culprit plaque rupture. One possible mechanism was that patients without PIA presented a higher incidence of STEMI onset upon exertion and physical exertion was a trigger of plaque rupture. In addition, Dai et al. have reported layered plaques, healing of ruptured plaque, were detected in 74.5% of patients with AMI and patients with layered plaques more often had PIA (39), indicating that the onset of AMI is dynamic and often preceded by plaque instability and thrombus formation for days or weeks (40). Therefore, it can be inferred that inflammatory response, thrombosis, and fibrinolysis might occur earlier in patients with PIA, which might affect the plaque morphology. However, no significant differences were observed in the prevalence of healed plaque between the two groups in this study, which might attribute to the lack of identification and detection of non-culprit plaques.

Notably, STEMI patients without PIA were at an increased risk of developing plaque rupture in the culprit lesion and had poor prognoses. These findings suggest that optimized care measures and customized treatment strategies are needed in the prehospital triage.

We also proved that reduced eGFR was an independent predictor of plaque rupture, implying that patients with renal insufficiency were at high risk of cardiovascular disease. Notably, Nakano et al. showed that chronic kidney disease was significantly associated with the severity of coronary atherosclerosis (41). Our finding is in line with a study from Kato et al., who showed that eGFR was insensitive for predicting coronary plaque vulnerability (42).

### Preinfarction Angina and Prognosis

Findings from previous studies assessed that the short- and long-term prognostic effects of PIA were conflicting. Schmidt et al. have demonstrated that PIA reduced 30-day mortality, particularly when unstable angina closely preceded AMI (4). In a large, prospective cohort (*n* = 16,439), ischemic presentations closer in time to AMI were reported to be related to lower 7-day mortality, but not long-term mortality (43). In contrast, Kobayashi et al. reported that patients with PIA had less severe AMI, smaller infarction size, and more favorable long-term survival (21). In addition, previous studies have confirmed that PIA was independently associated with lower 5-year mortality in patients with STEMI-PCI (3, 44). In this study, we reported that PIA was associated with lower MACE risk when 10 months after STEMI. Multiple mechanisms may explain the cardioprotective effects of PIA. First, PIA may act as a clinical surrogate of ischemic preconditioning (45) that may explain its prognostic value in lower MACE risk. Moreover, patients with PIA showed lower peak troponin I levels; the results supported a prior study indicating that PIA was associated with smaller infarct size (2). Because PIA is relatively common, it is important that these patients can be identified in more clinical trials. However, due to the small sample size and short follow-up time of this study, further studies on the prognostic value of PIA are needed.

### Clinical Implication

To the best of our knowledge, this study is the first study to demonstrate the absence of PIA as an independent predictor of plaque rupture in patients with STEMI. This study identifies the association between *in-vivo* culprit lesion morphologies and anticipatory angina, and demonstrates the linkage of clinical presentation and pathophysiological mechanisms. Considering the need for personalized and precise treatment strategies, more attention should be paid to individuals with a sudden onset of STEMI.

## Study Limitations

This study has several limitations. First, it is a single-center study with strict inclusion criteria, which resulted in a potential selection bias and the high proportion of patients with plaque erosion included in this study. Therefore, further investigations involving a large, multicenter study population are warranted. Second, the identification and diagnosis of PIA were based on the subjective descriptions of symptoms from patients, which might cause a confounding bias of our group classifications. Third, in some of the enrolled patients, it is ineluctable that the underlying plaque morphology may have been obscured due to the residual thrombi, although sufficient pre-OCT thrombus aspiration was performed and massive thrombus cases after aspiration were excluded. In addition, most patients in this study were not tested for creatine kinase (CK) and creatine kinase-MB (CK-MB) multiple times to gain the maximum levels of CK and CK-MB; thus, the current results could not fully reflect the relationship between PIA and infarct size. Finally, we cannot exclude the possibility that thrombus aspiration and predilatation prior to OCT imaging, despite being carried out with caution, might contribute to plaque rupture to some extent. Therefore, the potential effect of the pre-OCT operation must be given a serious consideration.

## Conclusion

Compared with patients without PIA, STEMI patients with PIA showed a significantly lower prevalence of plaque rupture and LRP in the culprit lesion, implying a different mechanism of AMI attack in these two groups of patients.

## Trial registration number

This study is registered at clinicaltrials.gov as NCT03593928 and Chinese National Natural Science Foundation (81970308).

## Data Availability Statement

The datasets used and analyzed during this study are available from the corresponding author on reasonable request. Requests to access these datasets should be directed to Hanjun Zhao, hbyanfuwai2018@163.com.

## Ethics Statement

The studies involving human participants were reviewed and approved by the Ethics Committee of Fuwai Hospital (No. 2017-866). The patients/participants provided their written informed consent to participate in this study.

## Author Contributions

YW and ZS: conception, data management, statistic, interpretation of data, and manuscript writing. HY: administrative support. HY, HZ, and LS: provision of study materials or patients. JL,YT, RC, XZ, and JZ: data interpretation and proofreading the manuscript.

## Funding

This study was funded by Chinese Academy of Medical Sciences Innovation Fund for Medical Sciences (No. 2016-I2M-1-009), National Natural Science Foundation of China (No. 81970308), Sanming Project of Medicine in Shenzhen (No. SZSM201911017), and Shenzhen Key Medical Discipline Construction Fund (No. SZXK001).

## Conflict of Interest

The authors declare that the research was conducted in the absence of any commercial or financial relationships that could be construed as a potential conflict of interest.

## Publisher's Note

All claims expressed in this article are solely those of the authors and do not necessarily represent those of their affiliated organizations, or those of the publisher, the editors and the reviewers. Any product that may be evaluated in this article, or claim that may be made by its manufacturer, is not guaranteed or endorsed by the publisher.
