# Supplementary material for: Association Between Preinfarction Angina and Culprit Lesion Morphology in Patients With ST-Segment Elevation Myocardial Infarction: An Optical Coherence Tomography Study
Source: Front Cardiovasc Med. 2022 Jan 18;8:678822. doi: 10.3389/fcvm.2021.678822 (PMC8804379; doi:10.3389/fcvm.2021.678822)
Supplement: Supplementary file 1 [file Table_1.DOCX]

**Supplemental Table 1.** Comparisons of Characteristics of Included and Excluded Patients

| **Variables** | **Included patients**  **(n = 279)** | **Excluded patients**  **(n = 155)** | **P value** |
| --- | --- | --- | --- |
| Men | 229 (82.1) | 137 (88.4) | 0.098 |
| Age, years | 57.3 ± 11.5 | 59.4 ± 11.8 | 0.081 |
| BMI, kg/m^2^ | 26.1 ± 3.7 | 26.2 ± 3.4 | 0.910 |
| Smoking | 194 (69.5) | 120 (77.4) | 0.093 |
| Hypertension | 163 (58.4) | 101 (65.2) | 0.183 |
| Diabetes | 95 (34.1) | 46 (29.7) | 0.393 |
| Dyslipidemia | 253 (90.7) | 146 (94.2) | 0.269 |
| Laboratory data |  |  |  |
| Total cholesterol, mg/dL | 167.05 (141.92, 196.83) | 160.87 (134.96, 186.39) | 0.014* |
| Triglyceride, mg/dL | 123.96 (79.69, 178.86) | 118.65 (81.46, 174.43) | 0.286 |
| LDL-C, mg/dL | 106.34 (85.07, 127.61) | 95.13 (73.86, 122.20) | 0.004* |
| HDL-C, mg/dL | 40.99 (35.58, 47.18) | 42.92 (34.80, 48.34) | 0.378 |
| Serum creatine, mmol/L | 78.6 (67.3, 90.9) | 81.7 (70.6, 91.3) | 0.162 |

Continuous data are presented as mean ± standard deviation or median (25^th^, 75^th^ percentile). Categorical data are presented as number (%). *P < 0.05. BMI, body mass index; HbA1c, haemoglobinA1c; HDL-C, high density lipoprotein cholesterol; LDL-C, low density lipoprotein cholesterol. (Adapted from reference (40) with permission).
