# Supplementary material for: Association Between Preinfarction Angina and Culprit Lesion Morphology in Patients With ST-Segment Elevation Myocardial Infarction: An Optical Coherence Tomography Study
Source: Front Cardiovasc Med. 2022 Jan 18;8:678822. doi: 10.3389/fcvm.2021.678822 (PMC8804379; doi:10.3389/fcvm.2021.678822)
Supplement: Supplementary file 2 [file Table_2.DOCX]

Supplementary Table 2. Baseline and OCT characteristics

|  | | | **Angina lasting for 3-30 minutes and occurred ≥2 times within 1 week** | | | | | |  | |
| --- | --- | --- | --- | --- | --- | --- | --- | --- | --- | --- |
| **Variables** | | | **Yes**  **(n = 87)** | | | **No**  **(n = 192)** | | | **P value** | |
| Age, years | | | 55.6 ± 11.9 | | | 58.1 ± 11.3 | | | 0.089 | |
| BMI, Kg/m^2^ | | | 26.8 ± 3.6 | | | 25.8 ± 3.0 | | | 0.032* | |
| Men, n (%) | | | 70 (80.5) | | | 159 (82.8) | | | 0.635 | |
| Smoking, n (%) | | | 39 (65.0) | | | 104 (71.2) | | | 0.378 | |
| Medical history, n (%) | | |  | | |  | | |  | |
| Hypertension | | | 47 (63.5) | | | 88 (52.4) | | | 0.108 | |
| Dyslipidemia | | | 64 (86.5) | | | 149 (88.7) | | | 0.627 | |
| Diabetes mellitus | | | 22 (29.7) | | | 53 (31.5) | | | 0.778 | |
| Prior PCI | | | 6 (8.1) | | | 21 (12.5) | | | 0.317 | |
| LVEF at admission, % | | | 55.6 ± 5.9 | | | 54.8 ± 6.7 | | | 0.396 | |
| Laboratory findings | | |  | | |  | | |  | |
| White blood cells, 10^6^/L | | | 9.7 ± 2.8 | | | 10.3 ± 3.0 | | | 0.119 | |
| Hs-CRP, mg/L | | | 5.5 (2.8-10.2) | | | 6.3 (2.6-11.1) | | | 0.319 | |
| HbA1c, % | | | 6.4 ± 1.2 | | | 6.7 ± 1.6 | | | 0.148 | |
| TC, mg/dL | | | 168.2 (148.6-197.0) | | | 165.3 (138.6-194.0) | | | 0.147 | |
| TG, mg/dL | | | 137.3 (82.0-189.6) | | | 122.3 (83.5-173.9) | | | 0.382 | |
| LDL-C, mg/dL | | | 109.4 (91.2-132.4) | | | 103.2 (81.4-125.4) | | | 0.096 | |
| HDL-C, mg/dL | | | 41.4 (35.0-46.6) | | | 40.4 (36.0-47.3) | | | 0.832 | |
| Lipoprotein (a), mg/L | | | 180.0 (94.0-389.1) | | | 145.0 (68.0-360.0) | | | 0.366 | |
| troponin I, ng/ml | | | 1.2 (0.1-3.7) | | | 0.9 (0.1-5.5) | | | 0.747 | |
| Peak troponin I, ng/ml | | | 17.0 (7.2-37.0) | | | 25.5 (11.2-49.1) | | | 0.039* | |
| Culprit vessels, n (%) | |  | | |  | | | 0.702 | |  |
| LAD | | 45 (51.7) | | | 90 (46.9) | | |  | |  |
| LCX | | 9 (10.3) | | | 19 (9.9) | | |  | |  |
| RCA | | 33 (37.9) | | | 83 (43.2) | | |  | |  |
| LM disease | | 3 (2.0) | | | 3 (2.4) | | | 0.999 | |  |
| Coronary artery lesions, n (%) | |  | | |  | | | 0.512 | |  |
| SVD | | 22 (25.3) | | | 27 (22.0) | | |  | |  |
| DVD | | 27 (31.0) | | | 73 (38.0) | | |  | |  |
| TVD | | 38 (43.7) | | | 73 (38.0) | | |  | |  |
| Prior-PCI procedures, n (%) | |  | | |  | | |  | |  |
| Aspiration | | 56 (65.1) | | | 127 (66.1) | | | 0.867 | |  |
| Pre-dilation | | 70 (81.4) | | | 151 (78.6) | | | 0.600 | |  |
| Pre-TIMI flow ≤1 | | 56 (65.1) | | | 131 (68.2) | | | 0.609 | |  |
| Optical coherence tomography findings | | | | | | | | | |  |
| Plaque morphology, n (%) |  | | |  | | | 0.013* | | |  |
| Plaque rupture | 34 (39.1) | | | 106 (55.2) | | |  | | |  |
| Intact fibrous cap | 53 (60.9) | | | 86 (44.8) | | |  | | |  |
| Plaque type, n (%) |  | | |  | | | 0.008* | | |  |
| Lipid-rich plaque | 34 (39.1) | | | 108 (56.2) | | |  | | |  |
| Fibrous plaque | 29 (33.3) | | | 53 (27.6) | | |  | | |  |
| TCFA, n (%) | 20 (23.0) | | | 49 (25.5) | | | 0.650 | | |  |
| Calcification, n (%) | 40 (46.0) | | | 104 (54.2) | | | 0.205 | | |  |
| Macrophage, n (%) | 45 (51.7) | | | 107 (55.7) | | | 0.534 | | |  |
| Micro-vessels, n (%) | 14 (16.1) | | | 35 (18.2) | | | 0.664 | | |  |
| Cholesterol crystal, n (%) | 3 (3.4) | | | 19 (9.9) | | | 0.064 | | |  |
| Thrombus, n (%) | 84 (96.6) | | | 188 (97.9) | | | 0.681 | | |  |
| Minimal FCT, μm | 131.1 ± 97.0 | | | 116.5 ± 80.9 | | | 0.190 | | |  |
| Maximal lipid arc, ° | 303.6 ± 73.2 | | | 304.5 ± 71.6 | | | 0.919 | | |  |
| MLA, mm^2^ | 1.80 ± 0.60 | | | 1.90 ± 0.80 | | | 0.056 | | |  |

Continuous data are presented as mean ± standard deviation or median (25^th^, 75^th^ percentile). Categorical data are presented as number (%). * P < 0.05. OCT, optical coherence tomography; BMI, Body mass index; PCI, percutaneous coronary intervention; LVEF, left ventricular ejection fraction; HS-CRP, high-sensitivity C-reactive protein; HbA1c, Glycated hemoglobin A1c; TC, total cholesterol; TG, triglyceride; LDL-C, low-density-lipoprotein cholesterol; HDL-C, high-density lipoprotein-cholesterol; LAD, left anterior descending; LCX, left circumflex artery; RCA, right coronary artery; LM, left main coronary artery; SVD, single-vessel disease; DVD, double-vessel disease; TVD, three-vessel disease; TIMI, Thrombolysis in myocardial infarction; TCFA, thin-cap fibroatheroma; FCT, fibrous cap thickness; MLA, minimal lumen area.
