# Supplementary material for: Association Between Preinfarction Angina and Culprit Lesion Morphology in Patients With ST-Segment Elevation Myocardial Infarction: An Optical Coherence Tomography Study
Source: Front Cardiovasc Med. 2022 Jan 18;8:678822. doi: 10.3389/fcvm.2021.678822 (PMC8804379; doi:10.3389/fcvm.2021.678822)
Supplement: Supplementary file 3 [file Table_3.DOCX]

Supplementary Table 3. Baseline and OCT characteristics

| **Variables** | | | **Non-PIA**  **(n = 126)** | | | **Unstable PIA**  **(n = 134)** | | | **Stable PIA**  **(n = 19)** | | | **P value** | |
| --- | --- | --- | --- | --- | --- | --- | --- | --- | --- | --- | --- | --- | --- |
| Age, years | | | 58.0 ± 11.1 | | | 56.9 ± 12.0 | | | 56.5 ± 11.3 | | | 0.785 | |
| BMI, Kg/m^2^ | | | 26.2 ± 3.4 | | | 26.3 ± 3.1 | | | 26.5 ± 2.8 | | | 0.448 | |
| Men, n (%) | | | 104 (82.5) | | | 110 (82.1) | | | 15 (78.9) | | | 0.930 | |
| Smoking, n (%) | | | 84 (66.7) | | | 69 (69.7) | | | 9 (69.2) | | | 0.996 | |
| Medical history, n (%) | | |  | | |  | | |  | | |  | |
| Hypertension | | | 68 (54.0) | | | 66 (57.4) | | | 8 (53.8) | | | 0.889 | |
| Dyslipidemia | | | 116 (92.1) | | | 99 (86.1) | | | 13 (86.7) | | | 0.629 | |
| Diabetes mellitus | | | 49 (38.9) | | | 33 (28.7) | | | 6 (40.0) | | | 0.631 | |
| Prior PCI | | | 12 (9.5) | | | 9 (7.8) | | | 5 (33.3) | | | 0.013* | |
| LVEF at admission, % | | | 54.6 ± 6.6 | | | 55.6 ± 5.9 | | | 54.8 ± 6.7 | | | 0.396 | |
| Laboratory findings | | |  | | |  | | |  | | |  | |
| White blood cells, 10^6^/L | | | 10.9 ± 3.1 | | | 9.9 ± 3.0 | | | 10.6 ± 2.7 | | | 0.440 | |
| Hs-CRP, mg/L | | | 6.1 (2.1-11.0) | | | 5.6 (2.7-10.4) | | | 8.3 (3.6-11.9) | | | 0.181 | |
| HbA1c, % | | | 6.6 ± 1.6 | | | 6.5 ± 1.3 | | | 6.5 ± 1.6 | | | 0.647 | |
| TC, mg/dL | | | 169.2 (141.1-203.8) | | | 166.0 (143.6-192.1) | | | 162.0 (134.2-181.3) | | | 0.568 | |
| TG, mg/dL | | | 127.1 (83.5-187.9) | | | 123.6 (79.7-176.1) | | | 117.8 (105.4-178.5) | | | 0.699 | |
| LDL-C, mg/dL | | | 110.2 (84.7-127.6) | | | 104.4 (85.1-127.0) | | | 97.4 (79.3-118.7) | | | 0.607 | |
| HDL-C, mg/dL | | | 40.4 (34.8-48.0) | | | 41.6 (36.3-47.1) | | | 38.3 (34.4-40.0) | | | 0.204 | |
| Lipoprotein (a), mg/L | | | 156.0 (68.0-356.5) | | | 160.4 (67.2-376.1) | | | 120.0 (88.2-257.5) | | | 0.983 | |
| troponin I, ng/ml | | | 1.0 (0.1-5.8) | | | 10.8 (0.1-3.5) | | | 1.1 (0.3-10.9) | | | 0.309 | |
| Peak troponin I, ng/ml | | | 27.0 (12.1-51.9) | | | 16.8 (8.7-38.3) | | | 26.2 (8.9-39.6) | | | 0.031* | |
| Culprit vessels, n (%) | |  | | |  | | |  | | | 0.834 | |  |
| LAD | | 62 (49.2) | | | 65 (48.5) | | | 7 (36.8) | | |  | |  |
| LCX | | 13 (10.3) | | | 13 (9.7) | | | 3 (15.8) | | |  | |  |
| RCA | | 51 (40.5) | | | 56 (41.8) | | | 9 (47.4) | | |  | |  |
| LM disease | | 3 (2.4) | | | 5 (3.7) | | | 1 (5.3) | | | 0.546 | |  |
| Coronary artery lesions, n (%) | |  | | |  | | |  | | | 0.837 | |  |
| SVD | | 27 (21.4) | | | 35 (26.1) | | | 6 (31.6) | | |  | |  |
| DVD | | 48 (38.1) | | | 46 (34.3) | | | 6 (31.6) | | |  | |  |
| TVD | | 51 (40.5) | | | 53 (39.6) | | | 7 (36.8) | | |  | |  |
| Prior-PCI procedures, n (%) | |  | | |  | | |  | | |  | |  |
| Aspiration | | 76 (60.3) | | | 91 (68.4) | | | 11 (57.9) | | | 0.588 | |  |
| Pre-dilation | | 100 (79.4) | | | 107 (80.5) | | | 13 (68.4) | | | 0.463 | |  |
| Pre-TIMI flow ≤1 | | 86 (68.3) | | | 89 (66.9) | | | 14 (73.7) | | | 0.826 | |  |
| Plaque morphology, n (%) |  | | |  | | |  | | | <0.001* | | |  |
| Plaque rupture | 78 (61.9) | | | 52 (38.8) | | | 10 (52.6) | | |  | | |  |
| Intact fibrous cap | 48 (38.1) | | | 82 (61.2) | | | 9 (47.4) | | |  | | |  |
| Plaque type, n (%) |  | | |  | | |  | | | <0.001* | | |  |
| Lipid-rich plaque | 87 (69.0) | | | 53 (39.6) | | | 10 (52.6) | | |  | | |  |
| Fibrous plaque | 39 (31.0) | | | 45 (33.6) | | | 6 (31.6) | | |  | | |  |
| TCFA, n (%) | 38 (30.2) | | | 26 (19.4) | | | 5 (26.3) | | | 0.131 | | |  |
| Calcification, n (%) | 67 (53.2) | | | 66 (49.3) | | | 10 (52.6) | | | 0.746 | | |  |
| Macrophage, n (%) | 72 (57.1) | | | 69 (51.5) | | | 11 (57.9) | | | 0.628 | | |  |
| Micro-vessels, n (%) | 18 (14.3) | | | 28 (20.9) | | | 3 (15.8) | | | 0.367 | | |  |
| Cholesterol crystal, n (%) | 12 (9.5) | | | 10 (7.5) | | | 0 (0.0) | | | 0.346 | | |  |
| Thrombus, n (%) | 122 (96.8) | | | 131 (97.8) | | | 19 (100.0) | | | 0.827 | | |  |
| Minimal FCT, μm | 111.4 ± 78.1 | | | 131.5 ± 94.5 | | | 112.1 ± 72.3 | | | 0.111 | | |  |
| Maximal lipid arc, ° | 306.1 ± 68.3 | | | 302.1 ± 73.0 | | | 306.8 ± 89.6 | | | 0.804 | | |  |
| MLA, mm^2^ | 1.91 ± 0.68 | | | 1.80 ± 0.70 | | | 2.10 ± 0.70 | | | 0.102 | | |  |

Continuous data are presented as mean ± standard deviation or median (25^th^, 75^th^ percentile). Categorical data are presented as number (%). * P < 0.05. BMI, Body mass index; PCI, percutaneous coronary intervention; LVEF, left ventricular ejection fraction; HS-CRP, high-sensitivity C-reactive protein; HbA1c, Glycated hemoglobin A1c; TC, total cholesterol; TG, triglyceride; LDL-C, low-density-lipoprotein cholesterol; HDL-C, high-density lipoprotein-cholesterol; LAD, left anterior descending; LCX, left circumflex artery; RCA, right coronary artery; LM, left main coronary artery; SVD, single-vessel disease; DVD, double-vessel disease; TVD, three-vessel disease; TIMI, Thrombolysis in myocardial infarction; TCFA, thin-cap fibroatheroma; FCT, fibrous cap thickness; MLA, minimal lumen area.
