# Supplementary material for: Association Between Preinfarction Angina and Culprit Lesion Morphology in Patients With ST-Segment Elevation Myocardial Infarction: An Optical Coherence Tomography Study
Source: Front Cardiovasc Med. 2022 Jan 18;8:678822. doi: 10.3389/fcvm.2021.678822 (PMC8804379; doi:10.3389/fcvm.2021.678822)
Supplement: Supplementary file 4 [file Table_4.DOCX]

Supplementary Table 4. Clinical outcomes

| **Variables** | **PIA group**  **(n = 153)** | **Non-PIA group**  **(n = 126)** | **P value** |
| --- | --- | --- | --- |
| MACE, n (%) | 21 (13.7) | 30 (23.8) | 0.111 |
| All-cause death, n (%) | 1 (0.7) | 1 (0.8) | 0.881 |
| Myocardial infarction, n (%) | 5 (3.3) | 3 (2.4) | 0.671 |
| Stroke, n (%) | 5 (3.3) | 4 (3.2) | 0.995 |
| Unplanned revascularization  , n (%) | 10 (6.5) | 22 (17.5) | 0.0036* |

* P < 0.05. PIA, pre-infarction angina; MACE, major adverse cardiovascular events.
