# Supplementary material for: Association Between Preinfarction Angina and Culprit Lesion Morphology in Patients With ST-Segment Elevation Myocardial Infarction: An Optical Coherence Tomography Study
Source: Front Cardiovasc Med. 2022 Jan 18;8:678822. doi: 10.3389/fcvm.2021.678822 (PMC8804379; doi:10.3389/fcvm.2021.678822)
Supplement: Supplementary file 5 [file Table_5.DOCX]

Supplementary Table 5. Baseline and OCT characteristics

| **Variables** | | | **PIA group**  **(n = 156)** | | | **Non-PIA group**  **(n = 140)** | | | **P value** | |
| --- | --- | --- | --- | --- | --- | --- | --- | --- | --- | --- |
| Age, years | | | 57.1 ± 12.0 | | | 58.9 ± 11.3 | | | 0.189 | |
| BMI, Kg/m^2^ | | | 26.2 ± 3.1 | | | 25.7 ± 7.0 | | | 0.205 | |
| Men, n (%) | | | 128 (82.1) | | | 114 (81.4) | | | 0.890 | |
| Smoking, n (%) | | | 79 (69.3) | | | 73 (69.5) | | | 0.971 | |
| Medical history, n (%) | | |  | | |  | | |  | |
| Hypertension | | | 76 (57.1) | | | 69 (55.2) | | | 0.753 | |
| Dyslipidemia | | | 115 (86.5) | | | 112 (89.6) | | | 0.439 | |
| Diabetes mellitus | | | 39 (29.3) | | | 40 (32.0) | | | 0.641 | |
| Prior PCI | | | 15 (11.3) | | | 15 (12.0) | | | 0.857 | |
| LVEF at admission, % | | | 54.9 ± 6.1 | | | 55.0 ± 7.0 | | | 0.882 | |
| Laboratory findings | | |  | | |  | | |  | |
| White blood cells, 10^6^/L | | | 10.0 ± 3.0 | | | 10.3 ± 3.0 | | | 0.542 | |
| Hs-CRP, mg/L | | | 5.8 (2.7-10.7) | | | 6.4 (2.6-11.0) | | | 0.780 | |
| HbA1c, % | | | 6.5 ± 1.3 | | | 6.7 ± 1.7 | | | 0.360 | |
| troponin I, ng/ml | | | 0.9 (0.1-4.4) | | | 0.9 (0.1-5.9) | | | 0.479 | |
| Peak troponin I, ng/ml | | | 17.3 (8.6-38.4) | | | 27.0 (11.7-51.5) | | | 0.012* | |
| Culprit vessels, n (%) | |  | | |  | | | 0.725 | |  |
| LAD | | 72 (46.2) | | | 69 (49.3) | | |  | |  |
| LCX | | 16 (10.3) | | | 15 (10.7) | | |  | |  |
| RCA | | 68 (43.6) | | | 56 (40.0) | | |  | |  |
| Coronary artery lesions, n (%) | |  | | |  | | | 0.433 | |  |
| SVD | | 41 (26.3) | | | 28 (20.0) | | |  | |  |
| DVD | | 54 (34.6) | | | 51 (36.4) | | |  | |  |
| TVD | | 61 (39.1) | | | 61 (43.6) | | |  | |  |
| Prior-PCI procedures, n (%) | |  | | |  | | |  | |  |
| Aspiration | | 103 (66.5) | | | 91 (65.0) | | | 0.793 | |  |
| Pre-dilation | | 123 (79.4) | | | 113 (80.7) | | | 0.771 | |  |
| Pre-TIMI flow ≤1 | | 105 (67.7) | | | 95 (67.9) | | | 0.983 | |  |
| Plaque morphology, n (%) |  | | |  | | | <0.001* | | |  |
| Plaque rupture | 62 (39.7) | | | 78 (55.7) | | |  | | |  |
| Plaque erosion | 91 (58.3) | | | 48 (34.3) | | |  | | |  |
| Calcified nodules | 3 (1.9) | | | 14 (10.0) | | |  | | |  |
| Plaque type, n (%) |  | | |  | | | <0.001* | | |  |
| Lipid-rich plaque | 63 (41.2) | | | 79 (62.7) | | |  | | |  |
| Fibrous plaque | 51 (33.3) | | | 31 (24.6) | | |  | | |  |
| TCFA, n (%) | 31 (20.3) | | | 38 (30.2) | | | 0.057 | | |  |

Continuous data are presented as mean ± standard deviation or median (25^th^, 75^th^ percentile). Categorical data are presented as number (%). * P < 0.05. OCT, optical coherence tomography; PIA, pre-infarction angina; BMI, Body mass index; PCI, percutaneous coronary intervention; LVEF, left ventricular ejection fraction; HS-CRP, high-sensitivity C-reactive protein; HbA1c, Glycated hemoglobin A1c; LAD, left anterior descending; LCX, left circumflex artery; RCA, right coronary artery; SVD, single-vessel disease; DVD, double-vessel disease; TVD, three-vessel disease; TIMI, Thrombolysis in myocardial infarction; TCFA, thin-cap fibroatheroma.
